# Bidirectional relationships between cannabis use, anxiety and depressive symptoms in the mediation of the association with psychotic experience: further support for an affective pathway to psychosis

**DOI:** 10.1017/S0033291722002756

**Published:** 2023-09

**Authors:** Rajiv Radhakrishnan, Lotta-Katrin Pries, Gamze Erzin, Margreet ten Have, Ron de Graaf, Saskia van Dorsselaer, Nicole Gunther, Maarten Bak, Bart P. F. Rutten, Jim van Os, Sinan Guloksuz

**Affiliations:** 1 Department of Psychiatry, Yale University School of Medicine, New Haven, CT, USA; 2 Department of Psychiatry and Neuropsychology, School for Mental Health and Neuroscience, Maastricht University Medical Center, Maastricht, the Netherlands; 3 Department of Psychiatry, Ankara Diskapi Training and Research Hospital, Ankara, Turkey; 4 Department of Epidemiology, Netherlands Institute of Mental Health and Addiction, Utrecht, the Netherlands; 5 Department of Psychiatry, UMC Utrecht Brain Centre, University Medical Centre Utrecht, Utrecht, the Netherlands; 6 Department of Psychosis Studies, King's College London, King's Health Partners, Institute of Psychiatry, London, UK

**Keywords:** Affective dysregulation, bidirectional relationship, cannabis, environment, longitudinal cohort, mediation analysis, population survey, psychosis, risk factors

## Abstract

**Background:**

Empirical evidence suggests that people use cannabis to ameliorate anxiety and depressive symptoms, yet cannabis also acutely worsens psychosis and affective symptoms. However, the temporal relationship between cannabis use, anxiety and depressive symptoms and psychotic experiences (PE) in longitudinal studies is unclear. This may be informed by examination of mutually mediating roles of cannabis, anxiety and depressive symptoms in the emergence of PE.

**Methods:**

Data were derived from the second longitudinal Netherlands Mental Health Survey and Incidence Study. Mediation analysis was performed to examine the relationship between cannabis use, anxiety/depressive symptoms and PE, using KHB logit in STATA while adjusting for age, sex and education status.

**Results:**

Cannabis use was found to mediate the relationship between preceding anxiety, depressive symptoms and later PE incidence, but the indirect contribution of cannabis use was small (for anxiety: % of total effect attributable to cannabis use = 1.00%; for depression: % of total effect attributable to cannabis use = 1.4%). Interestingly, anxiety and depressive symptoms were found to mediate the relationship between preceding cannabis use and later PE incidence to a greater degree (% of total effect attributable to anxiety = 17%; % of total effect attributable to depression = 37%).

**Conclusion:**

This first longitudinal cohort study examining the mediational relationship between cannabis use, anxiety/depressive symptoms and PE, shows that there is a bidirectional relationship between cannabis use, anxiety/depressive symptoms and PE. However, the contribution of anxiety/depressive symptoms as a mediator was greater than that of cannabis.

## Introduction

Epidemiological studies have consistently shown an association between cannabis use and the emergence of psychosis (Di Forti et al., 2019a; 2019b; Pries et al., 2018). Mendelian randomization studies examining the association between genetic predisposition, cannabis use and psychosis have found a bidirectional relationship i.e. genetic vulnerability predisposes to earlier cannabis use and cannabis use predisposes to psychosis among those with genetic vulnerability (Gillespie & Kendler, 2021). Patients with psychosis also have high rates of cannabis use (Boydell et al., 2006). The reasons for these seemingly counterintuitive findings of greater risk of psychosis with cannabis use and high rates of cannabis use among patients with psychosis are not fully understood. One possible explanation for these discrepant findings is that cannabis use ameliorates anxiety and depressive symptoms while also increasing the risk for psychotic experiences (PE).

While it is difficult to test this hypothesis in patients who have already developed psychosis, a general population, longitudinal sample such as the Netherlands Mental Health Survey and Incidence Study-2 (NEMESIS-2), provides a unique opportunity to examine this question. Approximately 5–8% of the general population may report PE (Linscott & van Os, 2013). We have previously shown that anxiety and depressive symptoms increase the risk of PE in interaction with environmental risk factors such as cannabis use and childhood adversity, consistent with an ‘affective pathway’ to psychosis (Guloksuz et al., 2015; Pries et al., 2018; Radhakrishnan et al., 2019; van Os et al., 2020). The important role of an ‘affective pathway’ to psychosis is also supported by experimental and observational studies (Bird, Waite, Rowsell, Fergusson, & Freeman, 2017; Freeman et al., 2013a, 2013b; Garety, Kuipers, Fowler, Freeman, & Bebbington, 2001; Hanssen, Bak, Bijl, Vollebergh, & van Os, 2005) where anxiety, depressive symptoms and worry is associated with paranoia and anomalous perceptual experiences, as well as experience sampling studies showing that increased stress-reactivity and negative affect are associated with subsequent increase in PE (Kramer et al., 2014; Myin-Germeys & van Os, 2007). A network analysis of psychopathology showed that affective symptoms were on the pathway between environmental risk-factors such as cannabis use and psychosis expression (Isvoranu et al., 2017). More recently, depression was shown to contribute to greater than multiplicative effect to the association between polygenic risk of schizophrenia, psychosis and childhood trauma (van Os et al., 2020). Additionally, it has been shown recently in the NEMESIS-2 data, that a fixed-effects model yields reliable estimates when examining the causal association between cannabis use and psychosis, and that the direction of causality is *from* cannabis use *to* psychosis i.e. cannabis use precedes the onset of psychosis (van Os et al., 2021a, 2021b). The question then arises whether the causal association between cannabis use and PE is mediated by anxiety/depressive symptoms or whether the relationship is bidirectional.

The presence of a bidirectional relationship between cannabis use and anxiety/depressive symptoms in the relationship to PE can be examined using mediation analysis. In this regard, we sought to examine the mediational relationship between cannabis use, anxiety/depressive symptoms, and PE. We hypothesized that cannabis use mediates the relationship between anxiety/depressive symptoms and PE. We also examined whether anxiety/depressive symptoms mediated the relationship between cannabis use and PE.

## Method

### Study population

The Netherlands Mental Health Survey and Incidence Study-2 (NEMESIS-2) is a longitudinal cohort study comprising 4 waves that examines the prevalence, incidence, course, and consequences of psychiatric disorders in the Dutch general population. The first wave (T0) that was conducted from 2007 to 2009, enrolled 6646 participants (response rate 65.1%; average interview duration: 95 min). They were followed up at year 3 (T1), year 6 (T2), and year 9 (T3) with successive response rates of 80.4% (*n* = 5303; excluding those who deceased; interview duration: 84 min), 87.8% (*n* = 4618; interview duration: 83 min), and 87.7% (*n* = 4007; interview duration: 101 min), respectively. The study was approved by the Medical Ethics Review Committee for Institutions on Mental Health Care and written informed consent was obtained from participants at each wave. A multistage random sampling procedure was applied to ensure representativeness of the sample to the general population in terms of age (between the ages of 18 and 65 at baseline), region, and population density. All participants were required to be literate in Dutch. The Composite International Diagnostic Interview (CIDI) version 3.0 [de Graaf, Ormel, ten Have, Burger, & Buist-Bouwman, 2008; Alonso J, et al., 2004] and additional questionnaires were administered by non-clinician, trained interviewers during home visits. Additional details of NEMESIS-2 are provided elsewhere (de Graaf, Ten Have, & van Dorsselaer, 2010; de Graaf, ten Have, van Gool, & van Dorsselaer, 2012). Data from all four waves were utilized. While there was some attrition between T0 and T3, this was not significantly associated with any of the individual 12-month mental disorders at T0 after controlling for sociodemographic characteristics (Nuyen et al., 2020).

### Outcome measure: psychotic experiences (PE)

PE were assessed using a 20-item binary-response questionnaire based on CIDI 1.1 as used elsewhere (Pries et al., 2018; Radhakrishnan et al., 2019; van Nierop et al., 2015), since earlier CIDI versions were found not to be adequate for capturing positive psychotic symptomatology. The primary binary outcome was the presence of any self-report PE conforming to previous studies (Guloksuz et al., 2020; Pries et al., 2021, 2020; Radhakrishnan et al., 2019).

### Assessment of anxiety and depressive symptoms

Symptoms of anxiety disorders including social phobia, specific phobia, panic disorder, generalized anxiety disorder, agoraphobia without panic disorder and symptoms of depressive disorders were assessed using CIDI 3.0 at each visit (de Graaf et al., 2008). Conforming to previous publications, we used two binary variables for depressive and anxiety symptoms, respectively (e.g. considered present if participants experienced at least one of the CIDI 3.0 core symptoms of depressive (or anxiety) disorder at each time-point) (Pries et al., 2018; Radhakrishnan et al., 2019).

### Assessment of cannabis use

The frequency of cannabis use over the last year was assessed using the section Illegal Substance Use of the CIDI 3.0 at each visit as used previously (Radhakrishnan et al., 2019). If subjects reported cannabis use, they were rated on frequency of use on a scale of 1 (never) to 7 (every day). A binary variable (absent = ‘0’ and present = ‘1’) was constructed by using the cut-off value of once per week or more in the previous year, consistent with other studies (Pries et al., 2021, 2020; Radhakrishnan et al., 2019).

### Statistical analysis

All analyses were performed using Stata, version 16 (StataCorp., 2019). The level of significance (*α*) was set at 0.05. To establish mediation, the following assumptions should be met [detailed in Baron and Kenny (1986), Gelfand, Mensinger, and Tenhave (2009)]: (1) The independent variable should be associated with the outcome; (2) There should be a association between the independent variable and the mediator, and between the mediator and the outcome; (3) The independent variable, mediated through the mediator, should be associated with the outcome. For mediation, the total effect of the independent variable on the outcome should be, at least partly, explained by the mediator (indirect effect). In the mediation model, the direct effect of the independent variable should decrease.

Given that mediation requires temporal precedence from independent variable to mediator to outcome, thus requiring absence of the outcome at the previous wave *T–1*, PE incident at time point *T* was modeled as a function of independent variable and mediator assessed over the interval *T–1* to *T*, conforming to previous analyses conducted in the NEMESIS-2 (van Os et al., 2021a, 2021b).

**Fig. 1.**
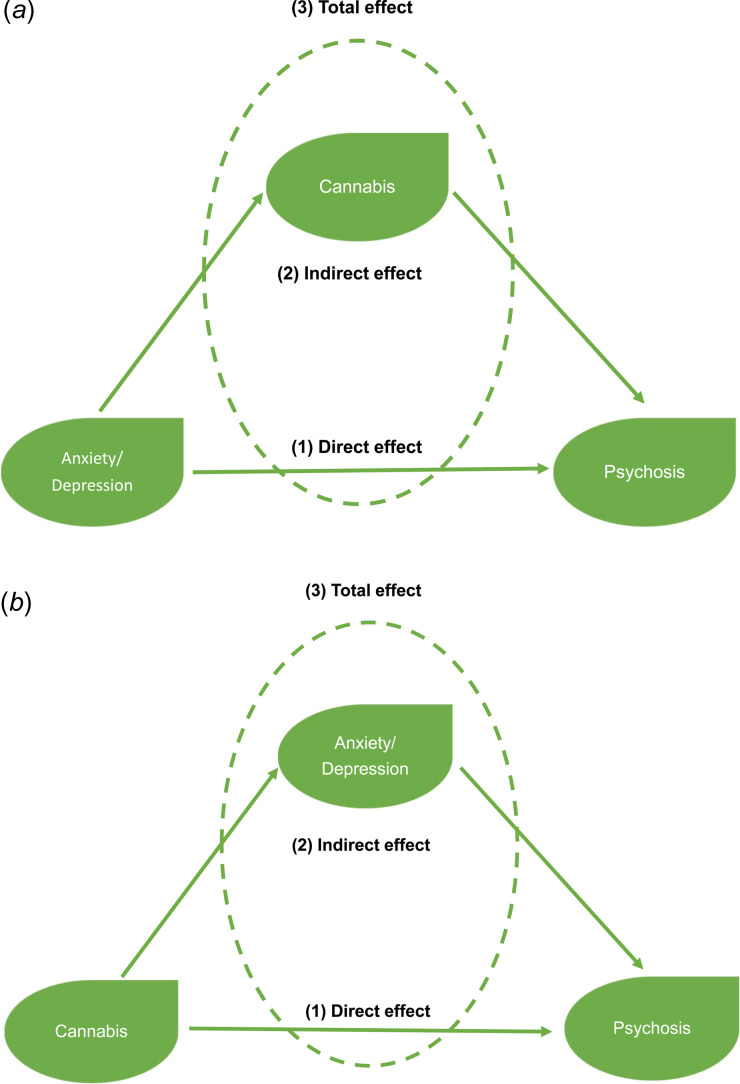
(*a*) and (*b*) illustrate hypothesized mediational model pairs. In (*a*) the model examines cannabis as a mediator of the effects of Anxiety/Depression on Psychosis outcomes. In (*b*), the model examines Anxiety/Depression as a mediator of the effects of Cannabis on Psychosis outcomes.

Logistic regression models were performed to analyze whether there was an association between the presence of affective dysregulation (any anxiety symptom and any depressive symptom, separately) at the preceding time point (*T–1*) and PE outcomes. Consequently, the KHB procedure, developed for estimating mediation in logistic regression models, was applied to estimate the mediation effect of cannabis use (Fig. 1*a*). Logistic regression models were performed to analyze whether there was an association between cannabis use in the previous year and PE outcomes. Subsequently, the KHB procedure was applied to estimate the mediation effect of the presence of affective dysregulation (any anxiety symptom and any depressive symptom, separately) (Fig. 1*b*). To correct for clustering of multiple observations within subjects, the CLUSTER option was applied in all logistic regression models to estimate cluster-robust standard errors.

All analyses were corrected for sex, age, education (1) primary school, (2) lower secondary education, (3) higher secondary education, (4) higher professional education).

## Results

This analysis used data from 6646 participants at four time points (T0, T1, T2, and T3) [*n* = 6646 at baseline (T0), *n* = 5303 in the first wave (T1), *n* = 4618 in the second wave (T2) and *n* = 4007 in the third wave (T3)]. Table 1 reports the demographics of the NEMESIS-2 at each time point.

**Table 1.** Demographics of the NEMESIS sample

	Baseline *n* = 6646	3-year follow up *n* = 5503	6-year follow-up *n* = 4618	9-year follow-up *n* = 4007
Female sex (%)	3672 (55.3)	2922 (55.1)	2558 (55.4)	2225 (55.5)
Mean age, years (s.d.)	44.3 (12.5)	47.6 (12.4)	50.9 (12.3)	54.1 (12.3)
Education				
Primary education (%)	332 (5)	226 (4.3)	186 (4)	154 (3.8)
Lower secondary education (%)	1826 (27.5)	1388 (26.2)	1193 (25.8)	1022 (25.5)
Higher secondary education (%)	2145 (32.3)	1728 (32.6)	1479 (32)	1274 (31.8)
Higher professional education, university degree (%)	2343 (35.3)	1961 (37)	1760 (38.1)	1557 (38.9)
Regular cannabis use in past year (%)	118 (2.2)	77 (1.5)	53 (1.2)	60 (1.5)
Presence of any anxiety/depression symptoms at each time point (%)^a^	3565 (53.6%)	1519 (27.6%)	1347 (29.2%)	1027 (25.6%)

Data are given as number (percentage) unless otherwise indicated.

a Baseline covers lifetime, and each follow-up covers the preceding period.

### Cannabis use as mediator for the association between anxiety symptoms and psychotic experience

Preceding anxiety symptoms were associated with cannabis use (OR 2.55, 95% CI 1.78–3.66, *p* < 0.001) and any self-reported PE (OR 1.99, 95% CI 1.65–2.42, *p* < 0.001); cannabis use was associated with self-reported PE (OR 2.36, 95% CI 1.25–4.46, *p* = 0.008). The mediation model revealed that cannabis use mediated the association between preceding anxiety symptoms and self-reported PE but explained only 1% of the relationship (Total effect of anxiety OR 1.97, 95% CI 1.63–2.39, *p* < 0.001; direct effect of anxiety OR 1.96, 95% CI 1.62–2.37, *p* < 0.001; indirect effect attributable to cannabis use OR 1.01, 95% CI 1.00–1.01, *p* = 0.045; % of total effect attributable to cannabis use = 1%) (Table 2).

**Table 2.** Results of decomposition of total effect, direct effect and indirect effect of the association between cannabis use, anxiety, and incidence psychosis using the Karlson–Holm–Breen (KHB) method

	Association between preceding anxiety and incidence psychosis with cannabis use as the mediator		Association between preceding cannabis use and incidence psychosis with anxiety as the mediator	
	OR (95% CI)	*p* value	OR (95% CI)	*p* value
Total effect (Reduced)	1.97 (1.63–2.39)	<0.001	1.75 (0.75–4.06)	0.195
Direct effect (Full)	1.96 (1.62–2.37)	<0.001	1.59 (0.68–3.71)	0.281
Indirect effect (Diff)	1.01 (1.00–1.01)	0.045	1.10 (0.99–1.21)	0.065
Confounding Ratio (total effect/direct effect)	1.01		1.20	
Confounding % (attributable to the mediator)	1.00		16.66	
Pseudo *R*^2^	0.02		0.04	

### Anxiety symptoms as mediator for the association between cannabis use and psychotic experience

Preceding cannabis use was associated with anxiety (OR 1.55, 95% CI 1.00–2.39, *p* = 0.05) and, directionally and with greater effect size, any self-reported PE (OR 1.77, 95% CI 0.76–4.12, *p* = 0.187); anxiety was associated with self-reported PE (OR 3.05, 95% CI 2.44–3.82, *p* < 0.001). The mediation model revealed that anxiety explained 17% of the total relationship (Total effect of cannabis OR 1.75, 95% CI 0.75–4.06, *p* = 0.195; direct effect of cannabis OR 1.56, 95% CI 0.68–3.71, *p* = 0.281; indirect effect attributable to anxiety OR 1.10, 95% CI 0.99–1.12, *p* = 0.065; % of total effect attributable to anxiety = 17%) (Table 2).

### Cannabis use as the mediator for the association between depressive symptoms and psychotic experience

Preceding depression was associated with cannabis use (OR 3.39, 95% CI 2.37–4.86, *p* < 0.001) and self-reported PE (OR 1.89, 95% CI 1.56–2.28, *p* < 0.001); cannabis use was associated with self-reported PE (OR 2.02, 95% CI 1.06–3.85, *p* = 0.033). The mediation model revealed that cannabis use was found to be a significant mediator of the relationship between depression and self-reported PE, but explained only 1.4% of the relationship (total effect of depression OR 1.86, 95% CI 1.54–2.25, *p* < 0.001; direct effect of depression OR 1.84, 95% CI 1.52–2.23, *p* < 0.001; indirect effect attributable to cannabis use OR 1.01, 95% CI 1.00–1.02, *p* = 0.045; % of total effect attributable to cannabis use = 1.4%) (Table 3).

**Table 3.** Results of decomposition of total effect, direct effect and indirect effect of the association between cannabis use, depression, and incidence psychosis using the Karlson–Holm–Breen (KHB) method

	Association between preceding depression and incidence psychosis with cannabis use as the mediator		Association between preceding cannabis use and incidence psychosis with depression as the mediator	
	OR (95% CI)	*p* value	OR (95% CI)	*p* value
Total effect (Reduced)	1.86 (1.54–2.25)	<0.001	1.77 (0.82–3.85)	0.146
Direct effect (Full)	1.84 (1.52–2.23)	<0.001	1.44 (0.66–3.13)	0.359
Indirect effect (Diff)	1.01 (1.00–1.02)	0.045	1.23 (1.11–1.37)	<0.001
Confounding Ratio (total effect/direct effect)	1.01		1.58	
Confounding % (attributable to the mediator)	1.39		36.65	
Pseudo *R*^2^	0.02		0.04	

### Depressive symptoms as the mediator for the association between cannabis use and psychotic experiences

Preceding cannabis use was associated with depressive symptoms (OR 2.43, 95% CI 1.58–3.74, *p* < 0.001) and self-reported PE at a trend level (OR 1.84, 95% CI 0.86–3.98, *p* = 0.118); depressive symptoms was associated with self-reported PE (OR 2.86, 95% CI 2.27–3.60, *p* < 0.001). The mediation model revealed that depressive symptoms were found to be a significant mediator of the relationship between cannabis use and self-reported PE, and explained 37% of the relationship (Total effect of cannabis use OR 1.77, 95% CI 0.82–3.85, *p* = 0.146; direct effect of cannabis use OR 1.44, 95% CI 0.66–3.13, *p* = 0.359; indirect effect attributable to depressive symptoms OR 1.23, 95% CI 1.11–1.37, *p* < 0.001; % of total effect attributable to depressive symptoms = 37%) (Table 3).

## Discussion

In this analysis of the NEMESIS-2 dataset, a large longitudinal cohort among the general population, we examined the mediational relationship between cannabis use, anxiety/depression symptoms (i.e. affective dysregulation) and PE. Our analysis showed that the mediational relationship between cannabis use, anxiety/depressive symptoms (affective dysregulation) and PE was bidirectional i.e. anxiety/depressive symptoms mediated the relationship between cannabis use and PE; and cannabis use mediated the relationship between anxiety/depressive symptoms and PE. However, although the relationship was bidirectional, the contribution of affective dysregulation in mediating the relationship between cannabis use and PE over time was greater than the mediational contribution of cannabis. These results lend further support for the presence of an ‘affective pathway’ to psychosis (Guloksuz et al., 2015; Pries et al., 2018; Radhakrishnan et al., 2019; van Os et al., 2020).

This is the first study to our knowledge, to show a bidirectional mediational relationship between cannabis use, affective dysregulation and psychosis in a general population sample. The directionality of increased anxiety mediating the association between cannabis use and psychosis is consistent with human laboratory challenge studies using delta-9 tetrahydrocannabinol (THC), the primary psychoactive ingredient, which show that administration of THC results in a significant increase in anxiety and psychosis-like experience (Ganesh et al., 2020; Hindley et al., 2020). Interestingly, preceding anxiety (pharmacologically induced using the GABA inverse agonist, Iomazenil) has also been shown to increase the psychosis-like effects of THC (Radhakrishnan et al., 2015). In epidemiological studies, cannabis use has been associated with greater depression in the general population (Rabiee et al., 2020), and among first-episode psychosis (Elowe, Golay, Baumann, Solida-Tozzi, & Conus, 2020), consistent with the directionality of cannabis use resulting in greater depression in our analysis. Animal studies show that THC has biphasic effects, i.e. low-dose THC is anxiolytic while high-dose THC is anxiogenic al (Salviato et al., 2021). While this has not been shown in human studies, an anxiolytic and antidepressant response to cannabis at low doses may explain the directionality of preceding anxiety, depressive symptoms resulting in increased cannabis use. These results are also consistent with the seemingly contradictory clinical observation that cannabis use is associated with psychosis, and people with psychosis are also at high risk of cannabis use.

However, as noted earlier, the contribution of cannabis use as a mediator of the relationship between affective dysregulation and PE was small in our analysis. These results hence do not support a self-medication hypothesis, but rather suggest that cannabis use results in increased affective dysregulation, supporting an ‘affective pathway’ to psychosis (Radhakrishnan et al., 2019; van Os et al., 2020).

Our results are consistent with studies examining the interactive effects of psychosis risk using schizophrenia polygenic risk score (PRS-SZ) for psychosis, cannabis use and affective dysregulation (Di Forti et al., 2019a, 2019b; Guloksuz et al., 2019; van Os et al., 2020). van Os et al., recently found that the relationship between PRS-SZ and psychosis showed significant dependence on the concomitant presence of affective dysregulation in 2 independent cohorts, NEMESIS-2 and EUGEI (van Os et al., 2020). This relationship was particularly evident for delusional ideation but not for hallucinatory experiences (van Os et al., 2020). A similar relationship was also with childhood trauma, such that the relationship between PRS-SZ and childhood trauma showed significant dependence on the concomitant presence of affective dysregulation, and was particularly evident for delusional ideation (van Os et al., 2020). Cannabis-induced dysphoria was also shown to mediate the relationship between childhood trauma and psychotic experiences (Carlyle et al., 2021).

The common biological mechanism that may explain this relationship remains to be elucidated. Interestingly, cannabis use, childhood trauma and affective dysregulation result in impairment in hippocampal structure and function (Barch et al., 2020; D'Souza et al., 2020; Treadway et al., 2015; Yuan et al., 2020; Zeredo et al., 2019). Abnormalities in hippocampal microstructure have also been seen in schizophrenia using PET imaging (Radhakrishnan et al., 2021). One hypothesis is that hippocampal dysfunction, *via* the hippocampal-striatal-midbrain network, leads to aberrant signaling of salience (Modinos et al., 2020) and hence, may represent a common biological mechanism leading to delusion formation, especially in those with affective dysregulation (Rauschenberg et al., 2021).

### Strengths and limitations

These findings should be interpreted in the light of several strengths and limitations. In terms of strengths, the present study has the advantage of a large number of participants representative of the Dutch population (Linscott & van Os, 2010; van Nierop et al., 2012). The analysis was conducted among 6646 participants at four time points. The study incorporated thorough symptom assessments across diagnostic boundaries providing an opportunity to examine trans-syndromal psychopathology in the general population. One limitation of the study is that the assessment of cannabis use was based on a questionnaire and might be subject to recall-bias and under-reporting. Similarly, the assessment of anxiety and depressive symptoms were based on CIDI 3.0 rather than clinical scales that specifically assess the severity of anxiety and depression, such as. Additionally, the current analysis could not answer the question of whether within-subject effects were different than between-subject effects as noted earlier (van Os et al., 2021a, 2021b). Another limitation is that the average age of participants at baseline in our sample was 44.3 years. This can in part account for the relatively lower contribution of cannabis use on the outcome compared to that of anxiety and depression. The impact of cannabis use on psychosis risk is greater in adolescence and it decreases over time. The information regarding the potency of cannabis was also not available in this sample. Additionally, cannabis use was coded as a dichotomous variable which limits the range of this measure in identifying whether more frequent use has a higher impact on psychosis.

## Conclusion

In this large, general population sample, we found that the mediational relationship between cannabis use, anxiety/depressive symptoms (affective dysregulation) and PE is bidirectional. The contribution of affective dysregulation in mediating the relationship between cannabis use and PE was far greater than the mediational contribution of cannabis. This study provides further support for the existence of an ‘affective pathway’ to psychosis. Whether interventions addressing affective dysregulation among cannabis-using individuals can reduce the risk of the emergence of PE remains to be verified.
